# Actual prospects of using some types of larch growing in Kazakhstan in medicine

**DOI:** 10.25122/jml-2021-0373

**Published:** 2022-08

**Authors:** Galiya Sayakova, Assyl Boshkayeva, Galiya Ibadullayeva, Akzhonas Khamitova, Gulzeynep Begimova

**Affiliations:** 1 Department of Pharmaceutical and Toxicological Chemistry, Pharmacognosy and Botany, Asfendiyarov Kazakh National Medical University, Almaty, The Republic of Kazakhstan; 2 Department of Pharmaceutical Technology, Non-profit Stock Corporation KazNMU, Almaty, The Republic of Kazakhstan

**Keywords:** Fine-scaled larch (*Lárix Kaémpferi*), Siberian larch (*Lárix Sibirica*), larch needles, macroscopic analysis, authenticity

## Abstract

Local plants can save natural resources and be used as a source of biologically active compounds, which can be high-quality, effective, and safe ingredients for pharmacological or chemical industries. Therefore, this study aimed to investigate the properties of two medicinal plants – the fine-scaled larch (*Lárix Kaémpferi*) and Siberian larch (*Lárix Sibirica*), which are growing in the Republic of Kazakhstan. We compared the two types of larches according to botanical affiliation and species description. We studied the alcoholic extracts from *Lárix Kaémpferi* and *Lárix Sibirica* to determine their physical and chemical properties. The data on the chemical composition of extractive compounds were generalized and systematized. The authenticity of *Lárix Kaémpferi* and *Lárix Sibirica* was established by external, anatomical, and diagnostic signs in microscopic examination and qualitative reactions. Specific indicators and their norms for raw materials were identified. This is the standard for both types of larch and determines their quality. We experimented with grinding fineness for studied materials and tested the impurities and moisture content of raw materials, total ash, and ash insoluble in a 10% hydrochloric acid solution. Determination of heavy metals and radionuclides was also considered. The quality specifications were developed based on the standardization of fine-scaled larch and Siberian larch.

## INTRODUCTION

Increasing life expectancy and sustaining the population's health is, as always, one of the most prioritized areas of the world. The World Health Organization (WHO) notes that the correct treatment with medicinal plants helps prevent a number of diseases [1]. Biologically active substances from medicinal plants contained in natural pharmaceutical preparations reduce the development of many diseases associated with immunodeficiency syndrome [2, 3].

The study of chemical composition, one of the mandatory requirements for determining the quality of medicinal plant raw materials used for drug development, is essential for health care [4–6].

One of the promising medicinal plants in pharmaceutical practice is the genus of woody plants of the family “Pine” larch (lat. *Lárix*), one of the most widespread species of conifers. There are 20 varieties of larch species known worldwide and used for pharmaceutical purposes. The composition of larch species is diverse and includes groups of important biologically active substances. The main commercial sources of North American larch species are high molecular weight polysaccharides. The immunomodulatory effect of larch arabinogalactan obtained from *Lárix laricina* and *Lárix occidentalis* (North American *Lárix* species) was discovered, and more specifically, its role in resistance to colds infections [7, 8].

For the first time, the diterpene compound, isocembrol, which has the properties of a hormone that regulates plant growth, was found among the substances extracted from the biomass of larch (*Lárix Sibirica Ledeb*.) [9, 10]. Antioxidant activity and the ability to radically purge a hydrophilic extract from coniferous and deciduous species, as well as seven pure compounds: lignans, flavonoids, and pinosylvin, isolated from conifers, were found [11–13].

Thus, the range of application of the larch species growing in Kazakhstan has not been fully studied. This concerns the degree of coverage of domestic plant species cultivated in Kazakhstan.

Larch species growing in Kazakhstan are of great importance. The Kazakh name of Siberian larch is “Sibir Balkaragayy”. It is a tree up to 30 m tall, with a wide pyramidal crown. The trunk is often thickened conically at the base. The bark is brownish brown, fissured. Branches are thick, spaced, young, bare, shiny, and light straw color. There are 30–40 leaves in a bunch, 2–4 cm long, 0.1–1 mm wide, and obtuse. Anther spikelets are pale yellow, and young cones are reddish, pubescent with red hairs, mature light brown, ovoid, 2–4 cm long, and 2–2.5 cm short. Their scales are 20–40, ovoid or rounded with a rounded, flat-cut, or less often slightly notched edge. Seeds are winged, yellowish with dark stripes and specks, with a wing 8–17 mm long. It blossoms in May-June, seeds ripen in August-September, and it grows in mountain forests. In Kazakhstan, it can be found in Altai and Tarbagatai or the southernmost location in Saur. The largest larch plantations are located in the Kaskelen gorge, on the territory of the Kaskelen forestry enterprise. It gives excellent wood and resin; the bark is dyed and can be used for forestry in Northern and Central Kazakhstan [14, 15].

This research aimed to study fine-scaled larch and Siberian larch growing on Kazakhstan's territory to develop quality specifications for the State Pharmacopoeia of the Republic of Kazakhstan.

## Material and Methods

Two types of larch needles were used in this study: fine-scaled larch (*Lárix Kaémpferi*), also named Japanese, and Siberian larch (*Lárix Sibirica*). Pharmacognostic and pharmacopoeial methods of analysis were used to determine numerical indicators of the quality of plant materials. The same methods were used to analyze the numerical and quality indicators of tested plant materials.

First, the authenticity of medicinal plant raw materials and extracts was analyzed by external signs, anatomical and diagnostic signs of morphological groups of two types of larch, and chemical composition. We used the standards of the General Pharmacopoeia Article of the State Pharmacopoeia of the Republic of Kazakhstan (sections “Determination of the morphological groups of medicinal plants”: of the specifications “Leaves” and “Bark”) as well as qualitative reactions to biologically active substances [16].

Determination of flavonones, phenol-containing compounds, and other biologically active substances was conducted by physicochemical methods (column chromatography, thin layer chromatography, and UV spectrophotometry). We used Byelorussian Pharmacopoeia and “Numerical indicators of quality” [17], which contain outlines for plant raw materials of *Lárix* and regulated norms that conform with the normative document requirements of the State Pharmacopoeia of the Republic of Kazakhstan: determination of fineness of grinding; determination of impurity content; determination of extractive substances; determination of moisture; determination of total ash and ash insoluble in 10% hydrochloric acid solution, determination of the content of heavy metals and radionuclides.

The determination of heavy metals and radionuclides was carried out following the common techniques of the State Pharmacopoeia of the Republic of Kazakhstan ("Sanitary-epidemiological requirements for radiation safety") [17].

### Quality characteristics of the plant materials testing

To analyze the quality characteristics of the raw materials tested, alcoholic extracts were obtained using ethanol of different concentrations. The plant material was preliminarily treated with non-polar solvents to remove resins, essential oils, pigments, and fatty acids. The alcoholic extracts were evaporated to isolate the individual components.

To confirm the quality, a set of tests were carried out to determine the physical and chemical properties of the objects under study. The maximum absorption in the UV spectrum ranged from 280 to 289 nm, which served as evidence of the presence of phenol-containing compounds in the plant raw material. We used a spectrophotometric method in the UV region on a device from the Bekman brand, Germany.

1. Reaction of azo dye formation. When phenol-containing compounds react with a diazonium salt, an azo dye is formed. The combination is in the o- and n-positions with respect to the phenolic hydroxyl of our compound in an alkaline medium (pH=9.0–10.0). In a neutral medium, a red precipitate forms. At pH>10, the azo coupling reaction will not proceed due to the transition of the diazonium salt to the dihydrate salt. We used this reaction for quantitative determination by spectrophotometry (SF) and photo electro colorimetry (FEC) in the visible region of the spectrum. To do this, we added 3 mL of sodium hydroxide solution (0.1 mol/L) to 1 mL of the extract (from the needles of fine-scaled larch and Siberian larch), heated in a water bath, cooled, and mixed with 1 mL of a freshly prepared diazotized solution of sulfanilic acid. The result is a red-orange to cherry-red colored product. Preparation of a diazo-reactive 5 mL of sulfanilic acid solution (45 mL of concentrated hydrochloric acid was added to 4.5 g of sulfanilic acid, and the volume of the solution was adjusted to 500 mL of purified water) was added to a 100 mL volumetric flask, placed on ice, 2.5 mL of 10% sodium solution, nitrite and a small piece of zinc were added. The resulting mixture was left on ice for 5 minutes, then another 10 mL of 10% sodium nitrite solution was added, shaken for 5 minutes, and the volume of the solution was brought to the mark. The solution was kept on ice; if necessary, savings can be made to reduce the number of reagents used.
2. Reaction of chalcone formation. 3 mL of filtrate formed a yellowish color with 10% sodium hydroxide solution (presence of flavones, flavanones, and flavanols).
3. Reaction with iron oxide chloride (FeCl3). 3 mL of a 1% solution of iron oxide chloride was added to the alcohol extraction, and a dark green color appeared as a result (polyphenolic compounds).
4. Reaction with a solution of iron-ammonium alum helped us to determine tannins in the material following a result of the reaction of alcoholic extraction with a solution of iron-ammonium alum. The indicator was a dark green color formation – tannins.

### Extraction technology

For the allocation of the extractive substances, 3 g of crushed raw material passing through a sieve with a hole size of 1 mm was placed in a flask with a thin section, and 50 mL of extractant was added. The flask was closed with a stopper, weighed to the nearest 0.01 g, and left for 1 hour. Then the flask was put on the reflux condenser and heated, maintained at a slight boil for 2 hours. The flask was cooled, closed with a stopper, weighed, and the mass loss was replenished with the extractant. The contents of the flask were shaken thoroughly and filtered through a paper filter into a dry flask.

After that, 25 mL of the filtrate evaporated in a water bath in a dried and accurately weighed porcelain dish. The residue was dried in an oven at a temperature of 102+2.5℃ to constant weight, then cooled in a desiccator for 30 minutes and weighed.

### Physicochemical methods of analysis

We took a water-alcohol extraction obtained from the needles of fine-scaled larch (*Lárix Kaémpferi*) and Siberian larch (*Lárix Sibirica*), collected in 2019 from different growing areas as a study object. Chromatography was carried out on Silufol UV-254 plates with an aluminum substrate. The analyzed sample volume was 2 µL. In order to better detect flavonoid compounds, the following solvent systems were studied and applied in studies: n-butanol-glacial acetic acid-water (4:1:5); benzene-ethanol-glacial acetic acid (23:4:2); ethyl acetate-glacial acetic acid-water (38:8:8). For the development of flavonoids on chromatographic slides, the following detecting agents were considered: 5% alcohol solution of AlCl3, ammonia vapor, and development in UV light.

### Numerical indicators of quality

#### Determination of fineness

The studied plant raw materials of fine-scaled larch (*Lárix Kaémpferi*) and Siberian larch (*Lárix Sibirica*) were placed on sieves (sieves of different sizes) and carefully sieved on a tray with rotary movements, avoiding additional grinding. The sifted crushed raw material was considered according to the requirements of the State Pharmacopoeia of the Republic of Kazakhstan.

#### Determination of moisture

An analytical sample of fine-scaled larch and Siberian larch sifted through a sieve was mixed to a particle size of 10 mm, and then samples weighing 3 g were taken and weighed with an error of 0.01 g. Each sample was individually weighed and dried with a bottle lid and placed in a heated 100–105℃ drying oven. The first weighing of the Larch needles was carried out after 60 minutes. A constant mass was achieved when the difference between two subsequent weighings after 30 min of drying and 30 min of cooling in a desiccator did not exceed 0.01 g. The results of the moisture study showed that in fine-scaled larch (*Lárix Kaémpferi*), it is 13%, and in Siberian larch (*Lárix Sibirica*), 14%.

#### Determination of total ash

3 g of the crushed raw material under study (accurately weighed) of fine-scaled larch and Siberian larch was placed in a pre-calcined and accurately weighed porcelain crucible, and the raw material was evenly distributed along the bottom of the crucible. Then the crucible with the contents of the object of study was heated in a muffle furnace, allowing the raw material to burn at a high temperature. Then, the flame was increased and burned until ash was obtained. Calcination was carried out at 750℃ to maintain constant weight. After the end of calcination, the crucible was cooled in a desiccator and weighed.

#### Determination of ash insoluble in 10% HCl

To the remainder of the crucible obtained after burning the raw material of fine-scaled larch and Siberian larch, 15 mL of a 10% solution of hydrochloric acid was added, covered with a watch glass, and carefully heated for 10 minutes in a boiling water bath. 5 mL of purified hot water was added to the contents of the crucible, washing the watch glass with it. The liquid was filtered through an ashless filter. The filter with the residue was washed with hot water purified to a negative reaction to chlorides in washing water, transferred to the same crucible, then dried, incinerated, calcined as described above, and weighed. The constant mass was weighed, according to the requirements of the State Pharmacopoeia of the Republic of Kazakhstan, so that the difference between the subsequent weighed ones after 30 min of drying and 30 min of cooling in a desiccator should not exceed 0.0005 g.

### Scientific novelty

The main purpose of this research was to use available herbal raw materials grown in the Republic of Kazakhstan for use in the pharmaceutical industry. The results could allow us to determine the presence of biologically active compounds suitable for further use in medical and pharmaceutical practice. Consequently, we looked at the research on the needles of domestic larch trees.

## Results

Quality indicators of larch medicinal plant raw materials are little studied. Therefore, the quality standard has to be created to form the national standardization for the larch raw materials. Our research can add a supplement to resolving this task. The content of heavy metals and radionuclides, which determine the safety of raw materials and compliance with the requirements for the place of its preparation, was established. The definition of these parameters was carried out following the common techniques of the State Pharmacopoeia of the Republic of Kazakhstan (Vol. 1, 2.8) and the standard sanitary-epidemiological requirements for radiation safety.

These plants were compared in terms of geographical distribution and morphology. Table 1 demonstrates the difference in their comparison.

**Table 1:** Comparative Botanical Characteristics of Fine-scaled larch and Siberian larch [18].

General points	Fine-scaled larch (*Lárix Kaémpferi*)	Siberian larch (*Lárix Sibirica*)
	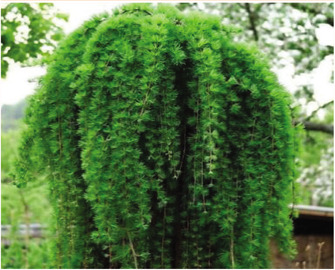	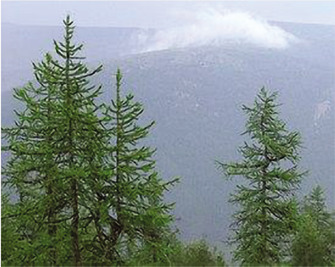
	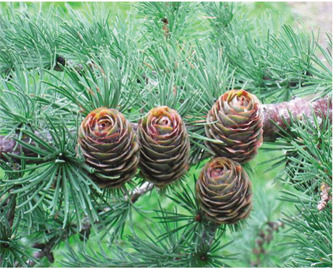	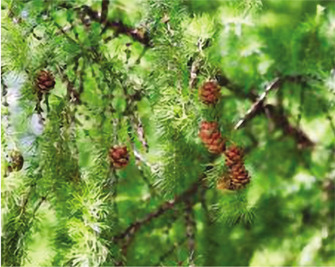
**Area of growth. Habitat**	Fine-scaled larch (*Lárix Kaémpferi*) (or Japanese) is a deciduous coniferous plant endemic to the island of Honshu and naturalized in Sakhalin. The tree also grows in Korea. It grows in the upper mountain forest belt at an altitude of 1600–2700 m above sea level. This plant also grows in Kazakhstan within the forest zone. It grows well in cold and dry climates. Among all species of larch, it is best tolerated by shading.	Siberian larch grows in the North-East of the European part of the countries of the Independent States, the Urals, and Western and Eastern Siberia. Distribution in Kazakhstan: found in Altai and Tarbagatai. The southernmost location in Saur. Some ecotypes of Siberian larch better tolerate heat and dryness, while others do not tolerate heat and dryness. For example, Siberian larch, which grows on the border of Kazakhstan's semi-desert, is sensitive to severe frosts. Many trees are found in the eastern part of Kazakhstan.
**Macroscopic analysis:** **species description**	Fine-scaled larch (*Lárix Kaémpferi*) (or Japanese) is a tall conifer of the Pine family (Pinaceae), reaching a height of up to 30 m. The crown is covered with blunt gray-emerald needles, reaching a length of 15 mm. The plant has a trunk with thin, peeling bark and long hanging branches slightly twisted in a spiral. At the beginning of winter, annual shoots acquire a brown-lemon color with blue bloom. Adult shoots are colored dark brown. Larch grows from a small seed, like the seeds of spruce and pine, equipped with a leathery wing. Larch seed is similar in wing shape to pine seed but is shorter and wider.	A mighty tree from the genus Larch (*Lárix*), of the Pine family (Pinaceae), up to 30–40m (grows in different ways in Kazakhstan, sometimes 10-20 m), with a trunk diameter of 50–100 cm (sometimes up to 2 meters). The crown of young trees is pyramidal. Later, after growing, it takes an oval-rounded shape. The bark on old trunks is grayish-brown, thick, with longitudinal cracks, and deeply furrowed. Larch branches are sparse, and the needles are not thick, soft, up to 50 mm long, 1.8 mm wide, with a blunt top. They are arranged in bunches on the shoots, and each bunch contains from 30 to 50 needles. In the fall, the needles, like other species of larch, fall off. Male spikelets (microstrobili) are single, spherical or oval, pale yellow, 5-6 mm in diameter, located at the ends of shortened shoots; female - broadly ovate-conical, 10-15 mm long, purple or pink, less often pale green or whitish. Pollination occurs in May.
**Distinctive features of the species**	Fine-scaled larch is a fast-growing plant, and the annual growth is 25 cm in height and 15 cm in width. In autumn, the needles are painted in a light lemon color, giving a decorative look.	Unlike coniferous trees, which are evergreen species, Siberian larch sheds all its foliage needles in autumn, which means it is a summer-green plant.
**Duration life of medicinal plant materials**	Fruiting occurs in the 15^th^ year of life. Larch is covered with round-oval cones 30 mm long, arranged in 5–6 rows. The fruits are formed from thin scales and can stay on the shoots for up to 3 years, forming light brown small seeds. Duration – up to 500–800 years.	The average lifespan of larch is 500–700 years, with specimens up to 800–900 years old.
**Typical properties of medicinal plant materials**	Fine-scaled larch (Kaémpferi) can grow in cold and dry climates, tolerates recurrent spring frosts, and is unpretentious in maintenance. Fine-scaled larch has strong wood; therefore, the plant is widely used in medicine and the woodworking industry. The need for use in medicine is because it has bactericidal properties, freshens the air, and drives away pests and parasites.	This plant is photophilous, not too changeable to the climate and soil, and resistant to weak spring frosts and low winter temperatures. It does not require a thermal regime during the growing season. Like fine-scaled larch, the wood of Siberian larch has bactericidal properties, refreshes the air, and drives away pests and parasites due to specific odors.
**Chemical composition**	Phytoncides are extracted from larch needles. The tinder fungus is found on larch trunks. The mushroom contains high concentrations of agaric acids, fats, phytosterol, mannitol and glucose, arabinogalactan (water-soluble polysaccharide). The medicinal properties of larch are due to a special chemical composition, determined by the content of carotene, lignin, glycosides, organic acids, anthocyanins, flavonoids, and gum. In young tender larch needles, the component composition of biologically active substances is determined by the content of ascorbic acid and essential oils (pinene, borneol, bornyl acetate etc). The component composition of the natural product, namely resin, is represented by essential oils and rosin, as well as phytoncides and the content of dihydroquercetin.	According to the literature data, Siberian larch is generally rich in useful substances: needles contain essential oil (0.18–0.20%), which includes α-pinene, borneol and bornyl acetate, ascorbic acid (0.2%), and an adhesive; bark – tannins (8-10%), coniferin glycoside, gum, catechins, flavonols, anthocyanins, organic acids; resin – essential oil and rosin (solid resin, represented by abietic acid); seeds – fatty drying oil (up to 18%). An essential oil (up to 16%) is obtained from the resin containing α-pinene, dipentene, sylvestrene, and α-sylvic acid. The fruiting body of the larch sponge is 60–65% composed of lipid substances soluble in ether. Various resins and organic acids are found in it, including agaricic, fatty oil etc. The plant contains flavanonols (from Latin flavus - yellow) – a group of flavonoids containing two asymmetric carbon atoms (at C-2 and C-3). Flavanonols do not contain chromophores, and therefore, as a rule, they are colorless. In medicinal plants, the most common dihydrokempferol and dihydroquercetin or taxi-folin (especially in Siberian larch wood).

Quality indicators were determined. The authenticity of the studied plant materials and the size and content of impurities were determined in the extracted samples of fine-scaled larch (*Lárix Kaémpferi*) and Siberian larch (*Lárix Sibirica*). The results for each species are shown in the tables. The results on the amount of extractive substances are shown in Table 2.

**Table 2:** The amount of extractive substances extracted from the medicinal plant raw materials.

Name of the investigated objects	The amount of biologically active substances					Normative documentation
	H_2_O	30% ethanol	50% ethanol	70% ethanol	96% ethanol	
**Fine-scaled larch (*Lárix Kaémpferi*)**	2.48	3.20	3.54	4.40	3.56	SPh RK (State Pharmacopoeia of the Republic of Kazakhstan). Vol. 1, section “Test methods for medicinal plant raw materials”.
**Siberian larch (*Lárix Sibirica*)**	2.20	3.0	3.30	4.20	3.40	

The flavanones from the raw material were extracted with ethyl acetate, n-butanol, and column chromatography (sorbent – aluminum oxide Al_2_O_3_, purified white powder). The results of the physical and chemical detection are presented in Table 3.

**Table 3:** Data of the qualitative detection of flavonoids in the needles of Fine-scaled (1) (*Lárix Kaémpferi*) and Siberian (2) (Lárix Sibirica) by the TLC method.

No.	(1) R_f_ (2) R_f_	Coloration of the spot after development in the visible area	Spot color developed in the UV region	Normative document
**Solvent system: n-butanol-glacial acetic acid-water (4:1:5)**				
**1**.	0.46 0.45	light yellow	yellow	SP RK, Vol. 1 – Thin-layer chromatography method (section 2.2.27)
**2**.	0.48 0.47	light yellow	yellow	
**3**.	0.54 0.52	light yellow	yellow	
**4**.	0.68 0.66	light yellow	yellow	
**5**.	0.52 0.64	light yellow	yellow	
**R_f_ medium 0.54 0.54**				
**Solvent system: benzene-ethanol-glacial a cetic acid (23:4:2)**				
**1**.	0.45 0.46	light yellow	yellow	SP RK, Vol. 1 – Thin-layer chromatography method (section 2.2.27)
**2**.	0.48 0.46	light yellow	yellow	
**3**.	0.68 0.65	light yellow	yellow	
**4**.	0.60 0.63	light yellow	yellow	
**5**.	0.58 0.60	light yellow	yellow	
**R_f_ medium 0.55 0.56**				
**Solvent system: ethyl acetate-glacial acetic acid-water (38:8:8)**				
**1**.	0.40 0.42	yellow-brown	brownish yellow	SP RK, Vol. 1 – Thin-layer chromatography method (section 2.2.27)
**2**.	0.42 0.42	yellow-brown	brownish yellow	
**3**.	0.44 0.45	yellow-brown	brownish yellow	
**4**.	0.47 0.46	yellow-brown	brownish yellow	
**5**.	0.38 0.36	yellow-brown	brownish yellow	
**R_f_ medium 0.42 0.42**				

The best detection of flavonoids is observed when using ammonia vapor. From the experimental data (Table 3), it can be seen that at least 5 flavonoids were found in the n-butanol-glacial acetic acid-water system. A solution of dihydroquercetin with a concentration of 0.5 mg/mL was chosen as a standard sample. The empirically obtained values of the retention factor (R_f_) for the standard sample solution (standard sample) of dihydroquercetin are presented in Table 4.

**Table 4:** Results of the detection of dihydroquercetin in the needles of fine-scaled larch (1) (*Lárix Kaémpferi*) and Siberian larch (2) (*Lárix Sibirica*) in various solvent systems.

No.	Solvent system	R_f_ the value of dihydroquercetin	Normative document
**1**.	N-Butanol-Glacial acetic acid-water (4:1:5)	0.54	SPh RK, Vol. 1 – Thin-layer chromatography method (section 2.2.27)
**2**.	Benzene-ethanol-glacial acetic acid (23:4:2)	0.56	
**3**.	Ethyl acetate-glacial acetic acid-water (38:8:8)	0.42	

In all the studied solvent systems, dihydroquercetin was identified by the R_f_ value and the nature of the resulting stains. According to the normative data, spot No. 3 identified in the benzene-ethanol-glacial acetic acid system is characteristic of flavonol-3-glycoside.

There is an influence of the studied raw materials size on the yield of biologically active substances and extractives. So, the permissible rate of the content of crushed particles for each type of larch is indicated in Table 5.

**Table 5:** Influence of the size of the studied raw materials on the yield of biologically active substances and extractives.

Name of the investigated objects	Crushing of raw materials in mm	Flavonoids in %: 70% ethanol 1:50 multiplicity 1, exposition 2 h	Extractive substances, %	Flavonoids in semples, %	Normative document
**Fine-scaled larch (*Lárix Kaémpferi*)**	1	10.71	10.71	10.71	State Pharmacopoeia of the Republic of Kazakhstan – determination of grinding (section “Test methods for medicinal plant raw materials”)
	2	17.22	17.22	17.22	
	3	14.84	14.84	14.84	
**Siberian larch (*Lárix Sibirica*)**	1	12.08	12.08	12.08	
	2	22.20	22.20	22.20	
	3	15.80	15.80	15.80	

We determined the impurity weight in the tested samples: each type of impurity was weighed on an analytical balance separately with an error of 0.1 g. The testing results are presented in Table 6.

**Table 6:** Numerical indicators of impurities in the needles of fine-scaled larch (*Lárix Kaémpferi*) and Siberian larch (*Lárix Sibirica*).

Name of objects	Benignity indicator	Set values	ND (normative document)
**Fine-scaled Larch (*Lárix Kaémpferi*)**	Browned parts	4.25	State Pharmacopoeia of the Republic of Kazakhstan (numerical indicators), section: general pharmacopoeial article 1.5.3.0004.15 (Determination of the authenticity, size and content of impurities in medicinal plant raw materials and herbal medicinal products)
	Organic impurities	0.62	
	Mineral impurities	0.84	
**Siberian Larch (*Lárix Sibirica*)**	Browned parts	4.20	
	Organic impurities	0.60	
	Mineral impurities	0.86	

Table 7 illustrates the results of the total ash of fine-scaled Larch (*Lárix Kaémpferi*) and Siberian larch (*Lárix Sibirica*).

**Table 7:** Determination of total ash of fine-scaled larch (*Lárix Kaémpferi*) and Siberian larch (*Lárix Sibirica*).

Name of objects	Benignity indicator	Set values	Normative document:
**Fine-scaled larch (*Lárix Kaémpferi*)**	Total ash in %	1.89	State Pharmacopoeia of the Republic of Kazakhstan (numerical indicators), section: general pharmacopoeial article 1.5.3.0004.15 ("Determination of the authenticity, size and content of impurities in medicinal plant raw materials and herbal medicinal products"
**Siberian larch (*Lárix Sibirica*)**	Total ash in %	2.50	

Furthermore, the ash insoluble in 10% HCl of fine-scaled larch (*Lárix Kaémpferi*) and Siberian larch (*Lárix Sibirica*) is presented in Table 8.

**Table 8:** Results of determination of ash insoluble in 10% HCl of fine-scaled larch (*Lárix Kaémpferi*) and Siberian larch (*Lárix Sibirica*).

Name of objects	Benignity indicator	Set values	Normative document:
**Fine-scaled larch (*Lárix Kaémpferi*)**	Ash, insoluble in 10% HCl	0.36	State Pharmacopoeia of the Republic of Kazakhstan (numerical indicators), section: general pharmacopoeial article 1.5.3.0004.15 ("Determination of the authenticity, size and content of impurities in medicinal plant raw materials and herbal medicinal products")
**Siberian larch (*Lárix Sibirica*)**	Ash, insoluble in 10% HCl	0.45	

Determination of the content of heavy metals and radionuclides in the objects under study are presented in Tables 9 and 10.

**Table 9:** Determination of heavy metals in plant raw materials of fine-scaled larch (*Lárix Kaémpferi*) and Siberian larch (*Lárix Sibirica*).

Name of the investigated objects	Toxic elements bq/kg, no more	Allowable norms for regulatory documents	Actual content	Test methods according to regulatory documents
**Fine-scaled larch (*Lárix Kaémpferi*)**	Lead	6.0	0.2980	State standard 17319-2019 State standard 30178-86
	Cadmium	1.0	0.0280	State standard 3078-96
	Arsenic	0.5	Not found	State standard 17319-2019 State standard 31266-04
	Mercury	0.1	Not found	State standard 26927
**Siberian larch (*Lárix Sibirica*)**	Lead	6.0	Traces	State standard 17319-2019 State standard 30178-86
	Cadmium	1.0	Not found	State standard 3078-96
	Arsenic	0.5	Not found	State standard 17319-2019 State standard 31266-04
	Mercury	0.1	Not found	State standard 26927

**Table 10:** Determination of radionuclides in plant raw materials of fine-scaled larch (*Lárix Kaémpferi*) and Siberian larch (*Lárix Sibirica*).

Name of the investigated objects	Radionuclides, bq/kg, no more	Allowable norms for regulatory documents	Actual content	Test methods according to regulatory documents
**Fine-scaled larch (*Lárix Kaémpferi*)**	Cesium-137	-	4.1	State standard 32161-2013 State standard 32163-2013
	Strontium-90	-	Not found	
**Siberian larch (*Lárix Sibirica*)**	Cesium-137	-	Not found	
	Strontium-90	-	Not found	

The content of heavy metals and radionuclides in the needles (*Lárix Kaémpferi*) is in line with the recommended norms.

Thus, the conformity of the quality of the researched plant raw materials belonging to the genus *Larix* was carried out according to external signs, anatomical and diagnostic features during microscopic examination of the types of raw materials, as well as qualitative reactions confirming the good quality of the types of raw materials. A comparative analysis of two plant species was carried out in the following sections: habitat, distribution, macroscopic analysis, species description, distinctive features of the species, life duration of medicinal plant materials, typical properties of medicinal plant materials, and chemical composition.

Quality indicators of authenticity and good quality were determined as a result of a pharmacognostic analysis of medicinal plant raw materials of *Lárix Kaémpferi* and *Lárix Sibirica*. Phytochemical analysis of larch trees confirmed the release of biologically active and extractive substances, depending on the degree of grinding of medicinal plant materials. Quality indicators were developed based on the standardization of fine-scaled larch and Siberian larch.

## Discussion

A comparative analysis of two types of larch was carried out: fine-scaled larch and Siberian larch, indicating the botanical affiliation and species description. Our results show that the quality of raw materials of widespread needles of fine-scaled larch (*Lárix Kaémpferi*) and Siberian larch (*Lárix Sibirica*) meets the requirements of the General Pharmacopoeia Articles of the State Pharmacopoeia of the Republic of Belarus, Kazakhstan. According to the results of the pharmacognostic analysis of medicinal plant raw materials of the fine-scaled larch and Siberian larch plants, qualitative indicators of the authenticity and good quality of the raw materials were determined. The research results showed that the quality of raw materials is a source and a qualitative basis for developing new herbal medicines suitable for the medical and pharmaceutical industries.

The research used pharmacognostic and pharmacopoeial methods to analyze the quantitative and qualitative indicators of the studied plant materials. Identification of medicinal plant materials by external signs and anatomical and diagnostic signs of morphological groups of larch species was carried out. The tests were carried out to determine the numerical indicators of the quality of raw materials: degree of grinding, impurity content: browned parts, organic impurities, and mineral impurities as recommended by State Pharmacopoeia of the Republic of Kazakhstan (numerical indicators, section: determination of the authenticity, size, and content of impurities in medicinal plant raw materials and herbal medicinal products); extractive substances; moisture; total ash and ash insoluble in 10% hydrochloric acid solution; heavy metals – in accordance with the requirements of the standards: State standard 17319 – 2019; State standard 30178-86; State standard 3078-96; State standard 31266-04; State standard 26927, as well as radionuclides – in accordance with the requirements of the standards: State standard 32161-2013; State standard 32163-2013.

To analyze the quality indicators of the tested raw materials, alcohol extracts were prepared, which were obtained from raw materials using ethanol of various concentrations.

The maximum absorption in the UV spectrum was from 280 to 289 nm, which served as evidence of the presence of phenol-containing compounds in the plant material. The proof of the content of phenol-containing compounds is a qualitative reaction (azo dye formation reaction). The presence of flavones, flavanones, and flavanols in medicinal plant materials was proven by the reaction of chalcone formation with the formation of a yellow precipitate.

Polyphenolic compounds were proven by reaction with iron oxide chloride. The content of tannins was confirmed by reaction with a solution of iron ammonium alum. The content of extractive substances during extraction with 70% alcohol was the highest percentage of the yield of biologically active substances among other extractants (water, 30% ethanol, 50% ethanol; 96% ethanol).

To better identify flavonoid compounds, chromatography was carried out in a thin layer of a sorbent with detection (using chemical agents (5% alcohol solution of AlCl3; ammonia vapor) and in the UV region (State Pharmacopoeia of the Republic of Kazakhstan: Vol. 1 – Thin-layer chromatography method (section 2.2.27)). The quantitative content of flavonoids ranges from 10.71% to 17.22% in *Lárix Kaémpferi* raw materials and from 12.08% to 22.20% in *Lárix Sibirica* raw materials.

### Practical application of research

The main types of larch are widely used in medical and pharmaceutical practice. In order to achieve a therapeutic effect, the needles are used to prepare a coniferous extract. Larch essential oil is used as turpentine ("Venetian turpentine"). The resin of Siberian larch and Siberian Fir (chewing resin or serka) is widely used for toothaches, inflammatory processes in the mouth and throat, tonsillitis and acute respiratory diseases, for the prevention of periodontal disease and caries, and for the restoration and strengthening of tooth enamel. Fine-scaled larch also has anti-inflammatory properties, so valuable substances (turpentine, rosin, acetic acid, sealing wax, tannins, essential oil, and much more) are obtained from larch wood processing products.

## Conclusion

The chemical composition of raw materials with a certain content of biologically active substances was confirmed. Flavonones, phenol-containing compounds, and other biologically active substances were determined by physicochemical methods (column chromatography, thin layer chromatography, and UV spectrophotometry).

All qualitative and quantitative indicators of the quality of raw materials constitute the content of quality specifications, considered private articles (monographs) for the State Pharmacopoeia of the Republic of Kazakhstan.

The results of the study of moisture showed that in *Lárix Kaémpferi*, it is 13%, and in *Lárix Sibirica*, it is 14%. Definition of total ash: for the fine-scaled larch (*Lárix Kaémpferi*) – 1.89%, for the *Lárix Sibirica* – 2.50%.

The content of heavy metals and radionuclides, which determine the safety of raw materials, was established and proved compliance with the requirements of the unified methods of the State Pharmacopoeia of the Republic of Kazakhstan (vol. 1, section 2.8) and regulatory regulations of sanitary and epidemiological requirements for radiation safety.

According to qualitative and quantitative indicators, a quality specification was developed for medicinal plant materials of the fine-scaled larch (*Lárix Kaémpferi*) and Siberian larch (*Lárix Sibirica*), which forms the structure of a private monograph of the State Pharmacopoeia of the Republic of Kazakhstan. These pharmacopoeial requirements form the content of the National Quality Standard for the standardization of medicinal plant materials of two types of Larch.
